# Necrotizing Sarcoid Granulomatosis: Another Great Imitator

**DOI:** 10.7759/cureus.85460

**Published:** 2025-06-06

**Authors:** Georgios Koukounides, Martin Ringel, Michael Osthoff, Manuel Schuster

**Affiliations:** 1 Department of Internal Medicine, Kantonsspital Winterthur, Winterthur, CHE; 2 Department of Pulmonology, Kantonsspital Winterthur, Winterthur, CHE

**Keywords:** atypical presentation of sarcoidosis, multiple pulmonary nodules, necrotizing sarcoid granulomatosis, pulmonary sarcoidosis, sarcoidosis treatment

## Abstract

Sarcoidosis with a necrotizing sarcoid granulomatosis (NSG) pattern is a rare disease that shares similar findings with sarcoidosis and is considered a distinct variant. However, the histological presence of necrosis may lead to misdiagnosis and a delay in treatment. We report the case of a 56-year-old Caucasian woman with cough, fatigue, dyspnea with lymphadenopathy, and consolidations on a CT scan. Histological examinations and a wide spectrum of diagnostic investigations, which excluded other infectious and non-infectious causes as well as malignancy, confirmed the diagnosis of sarcoidosis with an NSG pattern. Treatment with corticosteroids initially led to the regression of symptoms. However, the treatment had to be discontinued after a manifestation of corticosteroid-induced psychosis. The reintroduction of corticosteroids at a reduced dose was well tolerated and resulted in a good clinical and imaging response. Sarcoidosis with an NSG pattern must be distinguished from other systemic and infectious diseases. As emphasized in this report, a rigorous diagnostic workup is mandatory to establish the diagnosis. Although corticosteroids are considered the treatment of choice, possible side effects and dosage adjustments should be evaluated regularly.

## Introduction

Necrotizing sarcoid granulomatosis (NSG) has recently been termed sarcoidosis with an NSG pattern. It was first described in 1973 by the American pathologist A.A. Liebow [1], who speculated that this entity was a form of either angiocentric granulomatosis with a better prognosis or a variant of sarcoidosis. Due to the clinical features being similar to sarcoidosis, it has been suggested that NGS should be classified as a late stage of nodular sarcoidosis or a variant of sarcoidosis in which a vascular obstruction has led to necrosis, resulting in a histologically distinguished pattern [2-4]. Due to the non-specific clinical and radiological presentations mimicking many other conditions, even malignancy, diagnosis can be challenging. Although NSG most frequently affects the lungs, extrapulmonary manifestations are frequently described [4]. Although published data are limited, an analysis of the approximately 135 cases published showed that the disease occurs more frequently in women than in men and that the mean age of onset is 42 years [5]. If therapy is indicated, corticosteroids are usually the treatment of choice [5,6]. In some cases, a steroid-sparing therapy with, e.g., azathioprine, cyclophosphamide, or infliximab may be warranted, depending on organ manifestation and disease course [5-7].

## Case presentation

**Figure 1:**
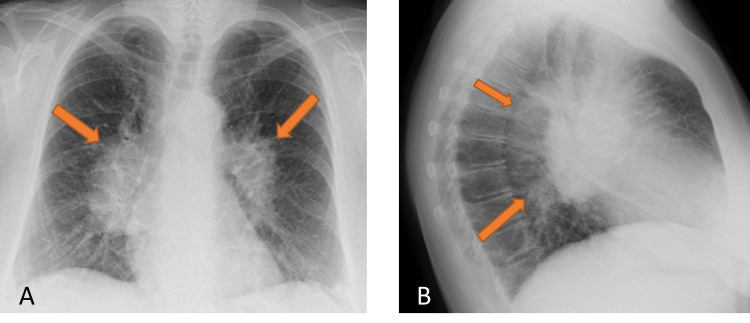
Chest X-ray showing a widened mediastinum (arrows). A. Posteroanterior view. B. Lateral view.

**Figure 2:**
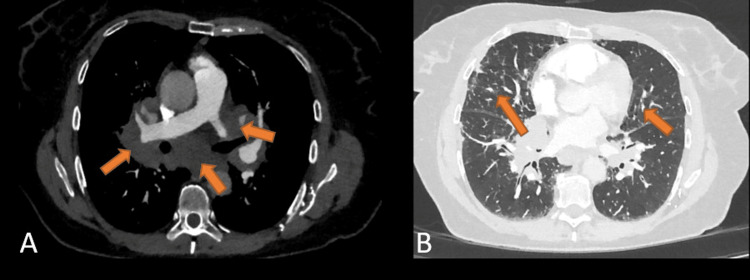
Initial CT of the thorax (axial view). A. Increased, enlarged, and confluent hilomediastinal lymph nodes (arrows). B. Bronchial thickening and interlobular septal thickening accentuated in both basal lobes, with peribronchovascular consolidations (arrows).

A 56-year-old, non-smoking female was referred by her general practitioner for further evaluation of a persistent non-productive cough, exertional dyspnea, fatigue, and weight loss of 10 kg for approximately six months. Due to a widened mediastinum in the initial chest X-ray (Figure 1), a contrast-enhanced CT scan was performed, revealing pronounced hilar and mediastinal lymphadenopathy, as well as interlobular septal thickening with diffuse subpleural consolidative changes and an 8 mm subpleural nodule in the right upper lobe (Figure 2). Furthermore, segmental pulmonary emboli of unknown age in the upper right lobe were also evident. Pulmonary function test demonstrated a moderate, non-reversible obstruction with normal diffusion capacity.

**Figure 3:**
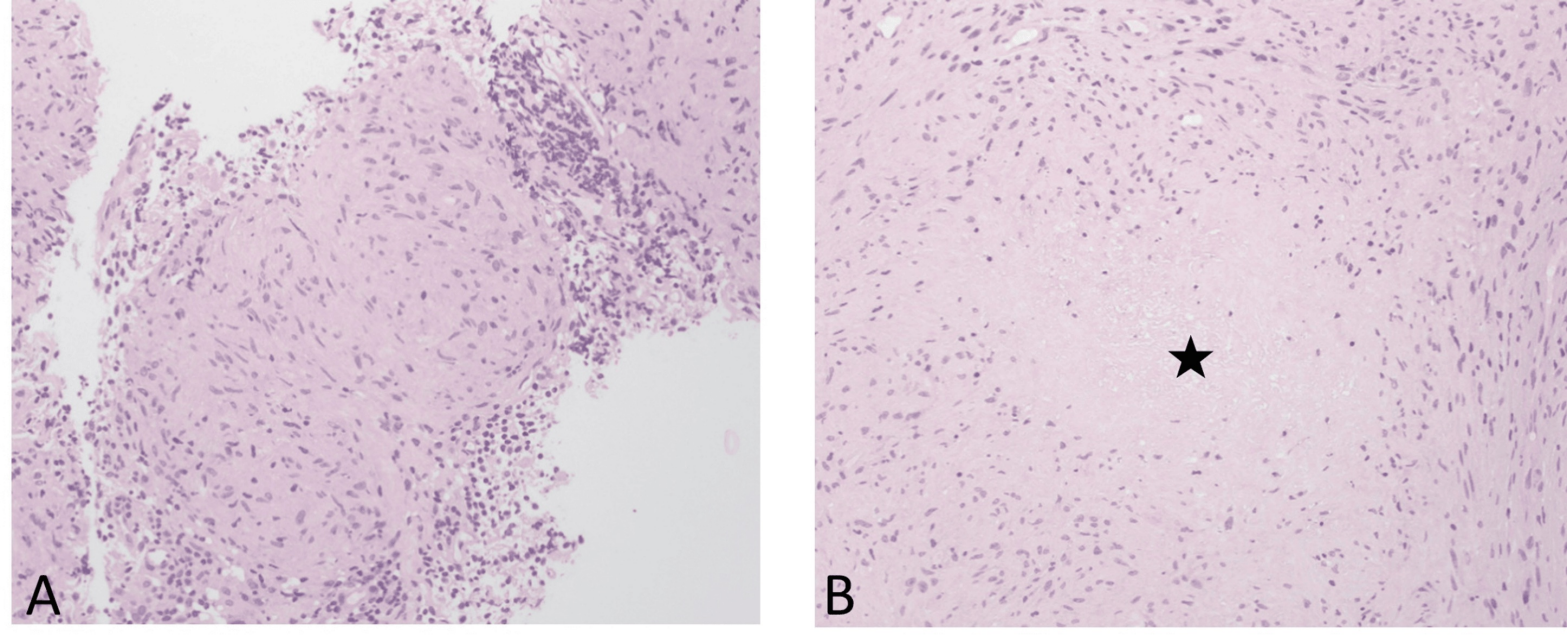
Cryobiopsy of the subcarinal lymph node (level 7). A. Epithelioid cellular granuloma without necrosis. B. Epithelioid cellular granuloma with central necrosis (★). Hematoxylin and eosin, original scale ×200.

For further diagnostics, bronchoscopy was performed promptly, and an endobronchial ultrasound-guided transbronchial needle aspiration as well as lymph node cryobiopsies were obtained. Histopathological analysis (Figure 3) revealed necrotizing epithelioid giant cell granuloma, raising suspicion of an infectious etiology. A standard bacterial culture and diagnostic testing for mycobacteria, including smear, polymerase chain reaction, and culture of the bronchial secretions, were all negative. Serological testing for IgG and IgM against *Francisella tularensis*, *Coxiella burnetii*, *Brucella* spp., *Bartonella henselae*, and HIV was all negative. Levels of angiotensin-converting enzyme activity and soluble interleukin-2-receptor were elevated (Table 1).

**Table 1:** Laboratory values of the patient, including microbiological analysis and reference values. ACE: angiotensin-converting enzyme; PCR: polymerase chain reaction

Laboratory tests	Reference values	Results
ACE activity	20–70 U/L	119.8 U/L
Soluble interleukin-2 receptor	<1,500 pg/mL	5,412 pg/mL
Serum immunology		
*Brucella* IgG	<20 U/mL	<5 U/mL
*Brucella* IgM	<15 U/mL	<5 U/mL
*Brucella* IgA	<10 U/mL	<5 U/mL
*Coxiella burnetii* phase II IgG	<20 U/mL	<5 U/mL
*Coxiella burnetii* phase II IgM	<0.9 MOC	0.2 MOC
*Francisella tularensis* IgG	<10 U/mL	<3 U/mL
*Francisella tularensis* IgM	<10 U/mL	<4 U/mL
*Bartonella henselae* IgG	1:<256	1:<128
HIV screening test	Negative	Negative
Tissue bacteriology		
PCR *Mycobacterium tuberculosis* complex	Negative	Negative
PCR Atypical mycobacteria	Negative	Negative
PCR *Bartonella quintana*	Negative	Negative
PCR *Bartonella henselae*	Negative	Negative
PCR *Francisella tularensis*	Negative	Negative
Mycobacterial culture	Negative	Negative

**Figure 4:**
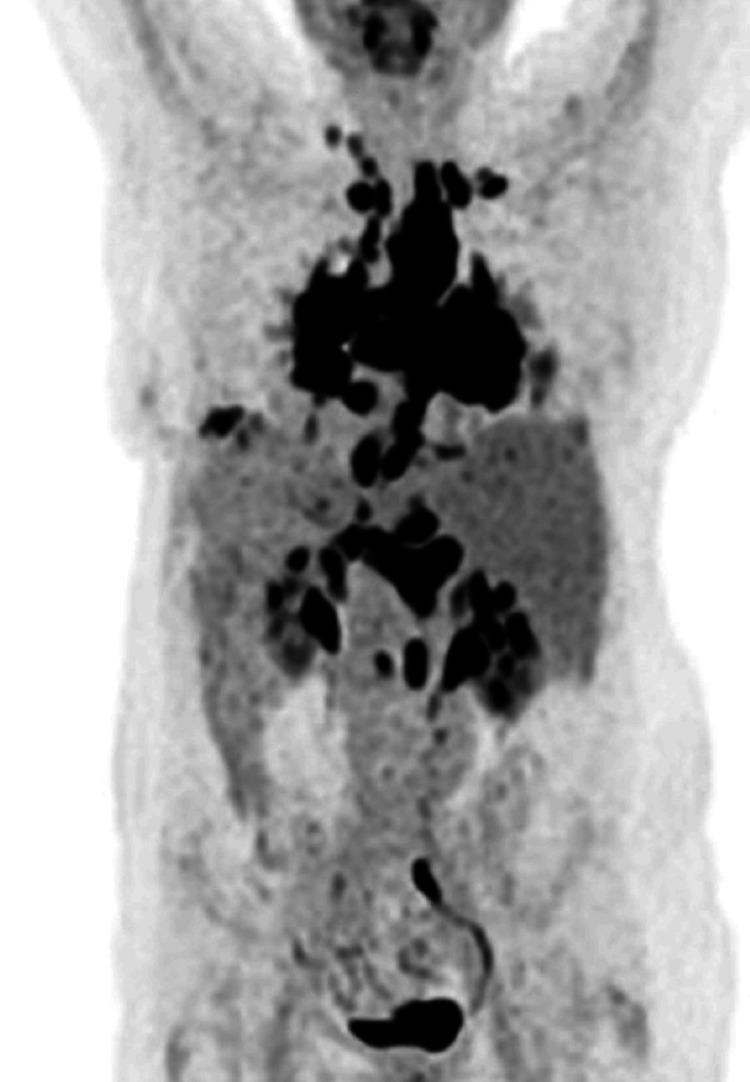
F18-fluorodeoxyglucose positron emission tomography/computed tomography scan (coronal view). The image shows intense metabolically active hilar, mediastinal, cervical/supraclavicular, upper abdominal, and retroperitoneal lymphadenopathy. Mild-to-severe pulmonary changes can be noted without evidence of cardiac involvement.

Given the high sensitivity and specificity of F18-fluorodeoxyglucose positron emission tomography/computed tomography (FDG-PET/CT) for the assessment of inflammatory activity, PET/CT was conducted to guide diagnosis and further management [8]. PET/CT (Figure 4) showed multiple enlarged cervical, mediastinal, hilar, retroperitoneal, and cardiophrenic lymph nodes with discrete or increased FDG-uptake (maximum standardized uptake value = 19.04, mean standardized uptake value = 12.32). Isolated septal and nodular changes were detected in the lung parenchyma, with the distribution pattern similar to sarcoidosis. There were no signs of further organ manifestations. Cardiac workup, including a 24-hour ambulatory electrocardiographic monitoring, transthoracic echocardiography, and PET/CT, showed no evidence of cardiac involvement.

Finally, a diagnosis of sarcoidosis with an NSG pattern was established. Given the patient’s symptoms and impaired lung function, steroid therapy with prednisolone at a dose of 40 mg daily was started. This resulted in the improvement of clinical symptoms within four weeks. Regarding lung function, forced expiratory volume in one second increased about 240 mL, achieving >80% of predicted value. A gradual tapering of the prednisolone therapy to 30 mg/daily, 20 mg/daily and 15 mg/daily after two, three, and four weeks, respectively, was performed.

Unfortunately, at the fourth week, the patient developed a psychotic disorder (delusions, distorted reality, restlessness, and anxiety), which started to manifest shortly after the initiation of prednisolone therapy according to the anamnesis noted by family members. Further diagnostic workup, including laboratory tests and MRI of the brain, excluded other causes, and a psychiatric evaluation led to the diagnosis of steroid-induced psychotic disorder. Therefore, hospitalization was necessary and treatment needed to be interrupted for approximately three weeks. Given the short duration of the therapy and the previous tapering, symptoms or signs of adrenal insufficiency were not observed at this time. Additionally, treatment with olanzapine was started and, soon after, psychiatric symptoms resolved completely. Considering the initially documented treatment response to steroid therapy, a cautious restart at a reduced dose of 10 mg/daily was initiated.

**Figure 5:**
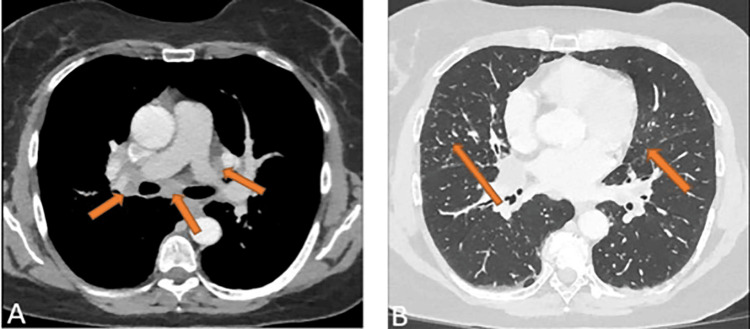
Thoracic CT at the ninth-month follow-up after the start of therapy (axial view). A. Compared to the previous CT scan, mediastinal and hilar lymph nodes (arrows) are smaller on both sides. B. Reduction in bronchial thickening, interlobular septal thickening, and subpleural consolidation (arrows).

On further follow-up, the patient reported an improvement in respiratory symptoms as well as her general condition while psychiatric side effects were absent. Laboratory analysis showed no evidence of inflammatory activity. At the ninth-month follow-up, chest CT showed a significant shrinkage in lymphadenopathy (Figure 5). Diffusion capacity and lung function showed normal dynamic lung volumes with a mild obstructive ventilation disorder, resulting in a gradual reduction in steroid therapy to 2.5 mg daily. Given the stable and overall positive course after two years of therapy, a timely therapy break with regular monitoring was planned.

## Discussion

Since the first description of the NSG by Liebow in 1973, subsequent reports have characterized it as a systemic disease with potential extrapulmonary involvement [9]. Although the disease has been reported earlier, it is still considered a rare finding with an undetermined incidence and is mainly a diagnosis of exclusion with a challenging approach [10].

Clinical presentations include non-specific symptoms such as fever, weight loss, night sweats, and fatigue, as well as pulmonary symptoms (e.g., dyspnea, chest pain), with a non-productive cough being described as the most frequent symptom [5,11]. Pulmonary function was not impaired in the majority of cases, although restriction and impaired diffusion capacity have been described [12]. Histological criteria for NSG pattern include granulomatous pneumonitis with sarcoid-like granulomas, fluctuating amounts of necrosis, and granulomatous vasculitis [9]. Although vascular involvement in sarcoidosis is reported frequently in autopsy studies and open-lung biopsy cases [13,14], necrosis in transbronchial biopsies is usually a hallmark of infectious (mycobacterial, fungal) granulomatosis [15]. Knowledge of this pattern is important to rule out sarcoidosis due to the presence of necrosis. Various infections, such as tularemia, that may cause similar manifestations with thoracic lymphadenopathy and granulomatosis should be evaluated, given local epidemiological data and patient exposure.

Radiological findings vary, and mostly solitary or multiple pulmonary nodules or masses are observed. Hilar and mediastinal lymphadenopathy has also been described in as many as 36% of the cases [5]. Further investigations, including PET/CT, can provide additional information and may suggest a sarcoidosis pattern, which may ultimately lead to the diagnosis [16].

Treatment decisions should be based on symptoms, organ involvement (especially cardiac), and the course of the function of the organ systems involved. Of note, as many as 38% of patients with NSG who received no treatment exhibited either complete or incomplete radiographic resolution of lesions on follow-up [17].

Although the initial treatment of choice is corticosteroids, as described in this case, serious adverse events are not uncommon. Given recent studies on sarcoidosis showing no inferiority of lower doses of prednisone (20 mg/day) in clinical efficacy, disease control, and quality of life, starting on a lower-dose regimen may be reasonable for sarcoidosis with an NGS pattern as well [18]. The use of corticosteroid-sparing agents can also be taken into consideration, such as in nodular sarcoidosis [7,19]; however, to date, there is a lack of data in sarcoidosis with an NSG pattern.

## Conclusions

We presented one of the very few published cases of a patient with sarcoidosis with an NSG pattern. Although rare, physicians should consider NSG in the diagnostic workup of patients presenting with necrotizing granulomatosis. After thorough exclusion of infection and malignancy, several features, such as response to glucocorticoids as well as matching histological and radiological findings, may lead to the diagnosis of this rare disease. Although the patient responded favorably to steroid therapy, treatment adjustment was ultimately required because of steroid-induced psychosis, which underscores the added value of this case report.
